# Potential application of human neural crest-derived nasal turbinate stem cells for the treatment of neuropathology and impaired cognition in models of Alzheimer’s disease

**DOI:** 10.1186/s13287-021-02489-1

**Published:** 2021-07-13

**Authors:** Jung Yeon Lim, Sang In Park, Soon A. Park, Jung Ho Jeon, Ho Yong Jung, Jung-Min Yon, Sin-Soo Jeun, Hyun Kook Lim, Sung Won Kim

**Affiliations:** 1 Department of Otolaryngology-Head and Neck Surgery, Seoul St. Mary’s Hospital, The Catholic University of Korea, 222 Banpo-Daero, Seocho-gu, Seoul, 06591 Republic of Korea; 2 Institute of Catholic Integrative Medicine (ICIM), Incheon St. Mary’s Hospital, The Catholic University of Korea, 56 Dongsu-ro, Bupyeong-gu, Incheon, 21431 Republic of Korea; 3 Department of Neurosurgery, Seoul St. Mary’s Hospital, The Catholic University of Korea, 222 Banpo-Daero, Seocho-gu, Seoul, 06591 Republic of Korea; 4 Department of Psychiatry, Yeouido St. Mary’s Hospital, The Catholic University of Korea, 63-ro 10, Yeoungdeungpo-gu, Seoul, 07345 Republic of Korea

**Keywords:** Alzheimer’s disease, hNTSCs, Neurogenic property, Cell transplantation, 5 × FAD mice

## Abstract

**Background:**

Stem cell transplantation is a fascinating therapeutic approach for the treatment of many neurodegenerative disorders; however, clinical trials using stem cells have not been as effective as expected based on preclinical studies. The aim of this study is to validate the hypothesis that human neural crest-derived nasal turbinate stem cells (hNTSCs) are a clinically promising therapeutic source of adult stem cells for the treatment of Alzheimer’s disease (AD).

**Methods:**

hNTSCs were evaluated in comparison with human bone marrow-derived mesenchymal stem cells (hBM-MSCs) according to the effect of transplantation on AD pathology, including PET/CT neuroimaging, immune status indicated by microglial numbers and autophagic capacity, neuronal survival, and cognition, in a 5 × FAD transgenic mouse model of AD.

**Results:**

We demonstrated that hNTSCs showed a high proliferative capacity and great neurogenic properties *in vitro*. Compared with hBM-MSC transplantation, hNTSC transplantation markedly reduced Aβ42 levels and plaque formation in the brains of the 5 × FAD transgenic AD mice on neuroimaging, concomitant with increased survival of hippocampal and cortex neurons. Moreover, hNTSCs strongly modulated immune status by reducing the number of microglia and the expression of the inflammatory cytokine IL-6 and upregulating autophagic capacity at 7 weeks after transplantation in AD models. Notably, compared with transplantation of hBM-MSCs, transplantation of hNTSCs significantly enhanced performance on the Morris water maze, with an increased level of TIMP2, which is necessary for spatial memory in young mice and neurons; this difference could be explained by the high engraftment of hNTSCs after transplantation.

**Conclusion:**

The reliable evidence provided by these findings reveals a promising therapeutic effect of hNTSCs and indicates a step forward the clinical application of hNTSCs in patients with AD.

## Background

Alzheimer’s disease (AD) is a devastating neurodegenerative disorder associated with a progressive decline in cognitive and memory function [1]. The pathological characteristics of AD include the accumulation of amyloid-β (Aβ) plaques and tau-laden neurofibrillary tangles, and progressive neuronal and synaptic loss [2]. However, there is currently no medication available to treat or prevent AD. Therapeutic approaches using stem cells for the treatment of neuropathology and loss of cognitive function in AD have become an important issue over the past few decades.

Mesenchymal stem cells (MSCs), including bone marrow-derived mesenchymal stem cells (BM-MSCs), umbilical cord blood-derived mesenchymal stem cells (UCB-MSCs), and adipose-derived mesenchymal stem cells (AD-MSCs), show promise for use in therapeutic and translational applications due to their high accessibility, relative ease of handling, potential to replace degenerating neurons, and neuroprotection induced via secretion of molecules that are crucial for therapy [3–5]. There is much evidence for the safety and efficacy of MSC therapies in animal models of AD. The transplantation of human BM-MSCs or UCB-MSCs into the hippocampus improves cognitive functions and modulates neuropathology in AD animal models [6–8], which has supported a handful of ongoing clinical trials of MSCs in patients with AD. Accumulating preclinical evidence suggests that MSCs have potential for treating AD; however, there is notably poor translation of these approaches from animal experiments to human clinical trials. Indeed, in human clinical trials, no patients showed any serious adverse events after transplantation of MSCs. However, clinical trials using MSCs were not as effective as expected based on preclinical studies; no changes to AD pathology or cognitive function were observed in MSC-treated patients [9]. Although stem cell-based therapy is certainly a promising approach for the treatment of AD, it is important to identify the characteristics of the stem cells to be used and optimize the cells to increase the therapeutic efficacy of stem cell application.

To obtain sufficient numbers of cells for clinical trials, it is necessary to repeat the expansion of cells several times in vitro, which causes the loss of multiple properties of stem cells. It has been demonstrated that BM-MSCs lose the ability to self-renew and differentiate into multiple lineage cells with expanded cell passaging [10]. Moreover, a major problem with the use of MSCs in brain disease is low engraftment and a poor ability to differentiate into neuronal lineage cells that retain the ability to replace damaged cells or tissue after transplantation [11, 12], which decreases the therapeutic effect of MSCs for the treatment of neurodegenerative disorders. Therefore, many studies have focused on finding alternative, valuable stem cell sources to overcome the limitations of MSCs and thus improve therapeutic efficacy in clinical applications.

Neural crest-derived stem cells (NCSCs) have been found in various adult tissues, such as the hard palate [13], oral mucosa [14, 15], cornea [16], periodontal ligament [17, 18], and inferior turbinate tissue [19, 20]. Recently, it has been reported that hNTSCs can be isolated from human nasal inferior turbinate tissue removed during turbinate resection, which is frequently performed to relieve nasal obstructions resulting from turbinate hypertrophy. hNTSCs can be obtained easily from human nasal inferior turbinate tissue obtained by minimally invasive collection procedures [21, 22]. Considering the frequency of turbinate surgery, it is possible to obtain a sufficient number of hNTSCs via in vitro expansion and provide hNTSCs for clinical application. hNTSCs showed a strong proliferation capacity and an ability to differentiate into multiple cell types in vitro and in vivo [21–24]. Moreover, hNTSCs exhibit preservation of multiple biological characteristics in culture, independent of donor and cell expansion conditions [22, 25], and do not cause safety issues in immunodeficient mice [24]. These considerations make hNTSCs a valuable alternative source of MSCs for tissue regeneration and clinical trials for the treatment of AD.

In this study, we investigated the possibility of the clinical application of hNTSCs for the treatment of AD by evaluating multiple pathological features associated with AD and cognitive recovery. Here, hNTSCs were transplanted intracranially in a 5 × FAD transgenic mouse model of AD, and then the effect of hNTSCs on cognition and disease pathology, including PET/CT neuroimaging, immune status indicated by microglial numbers and autophagic capacity, and neuronal survival, was assessed throughout a 6–7-week posttransplant period.

## Materials and methods

### hNTSC culture

The study procedure utilizing hNTSCs was conducted in compliance with the Institutional Review Board of Seoul St. Mary’s Hospital (KIRB-00631_4-008), Catholic University of Korea, as well as informed consent regulations, and the Declaration of Helsinki. Before surgery, the participants provided written informed consent to participate in this study. hNTSCs were isolated from discarded nasal inferior turbinate tissue from human patients who underwent partial turbinectomy, as previously described [21, 22]. The tissue was washed with a saline solution and phosphate-buffered saline (PBS; Thermo Fisher Scientific, Waltham, MA, USA) and cut into 1-mm^3^ small pieces, which were then plated in a culture dish and covered with a sterilized glass cover slide. The tissue was incubated at 37 °C in a humidified atmosphere containing 5% (v/v) CO_2_ in α-minimum essential medium (α-MEM; Thermo Fisher Scientific) supplemented with 1% (v/v) penicillin/streptomycin (antibiotics, Invitrogen, Carlsbad, CA, USA) and 10% (v/v) fetal bovine serum (FBS; Thermo Fisher Scientific). The culture medium was changed every 2 days during 3 weeks of culture. The glass cover slide was removed and cells isolated from the tissue were harvested using 0.25% trypsinin 1 mM EDTA solution. hNTSCs were expanded for use in experiments. To analyze cell growth in the culture, 9 × 10^3^ cells were seeded in 24-well culture plates and then measured for 4 days using an EZ-Cytox assay kit (DAEILLAB Co., Seoul, Korea, http://www.daeillab.co.kr). Absorbance at a wavelength of 450 nm was determined using a microplate reader (MolecularDevices Corporation, Sunnyvale, CA, USA).

### hBM-MSC culture

Human bone marrow aspirates were obtained from the iliac crest of healthy donors aged 20 to 55 years after approval by the Institutional Review Board of Seoul St. Mary’s Hospital (approval Nos. KIRB-00344-009 and KIRB-00362-006). Bone marrow aspirates from participants who provided written informed consent were sent to the good manufacturing practice-compliant facility of the Catholic Institute of Cell Therapy (Seoul, Korea, http://www.cic.re.kr) for the isolation, expansion, and quality control of hBM-MSCs, as previously described [26]. hBM-MSCs were cultured in Dulbecco’s modified Eagle’s medium (DMEM) supplemented with 1% (v/v) penicillin/streptomycin (Invitrogen) and 20% (v/v) FBS (Thermo Fisher Scientific). The cells were incubated at 37 °C in a humidified atmosphere containing 5% (v/v) CO_2_.

### Alizarin Red S staining

To analyze osteogenic differentiation, the cells were cultured in osteogenic differentiation medium for 3 weeks (PromoCell, Heidelberg, Germany). The cells were changed with fresh medium three times a week for 3 weeks. The cells were fixed with 4% (w/v) paraformaldehyde (PFA) on day 21 after culture, and deposition was detected by staining with 4% (w/v) Alizarin Red S solution (Sigma-Aldrich Co., St. Louis, MO, USA). The stained cells were observed by microscopy (ECLIPSE Ci-E, Nikon, Tokyo, Japan).

### Oil Red O staining

To analyze adipogenic differentiation, the cells were cultured in adipogenic differentiation medium for 3 weeks (Stem Pro®, Thermo Fisher Scientific). The cells were changed with fresh medium three times a week for 3 weeks. The constructs were fixed with 10% (w/v) formalin on day 20 after culture and lipid droplets were detected by staining with 0.5% (w/v) Oil Red O solution (Sigma-Aldrich). The stained cells were observed using an ECLIPSE Ci-E microscope (Nikon).

### Safranin O staining

To analyze chondrogenic differentiation, the cell pellets were cultured in chondrogenic differentiation medium for 2 weeks (PromoCell). The pellets were changed with fresh medium three times a week for 2 weeks. The pellets were fixed with 10% (w/v) formalin on day 14 after culture prior to paraffin embedding and sectioning. Chondrocytes were detected by staining with Safranin O solution (NovaUltra Safranin O stain kit, Thermo Fisher Scientific). The stained cells were observed by microscopy (ECLIPSE Ci-E, Nikon, Tokyo, Japan).

### Cell transplantation

For the in vivo study, mice expressing five mutants of human AβPP and PS1 (5 × FAD) (B6SJL-Tg[AβPP *K670N*M671L*I716V*V717I, PSEN1*M146*L286V]6799Vas/J) under the control of the Thy1 promoter (16 weeks of age, male; The Jackson Laboratory, Bar Harbor, ME., USA) were used in accordance with institutional guidelines under conditions approved by the Institutional Animal Care and Use Committee of The Catholic University of Korea. The mice were divided into four treatment groups (15 mice per group): (1) wild-type (WT) mice treated with PBS, (2) transgenic (Tg) mice treated with PBS, (3) Tg mice treated with hNTSCs, and (4) Tg mice treated with hBM-MSCs. Sixteen-week-old WT or TG mice were anesthetized with ketamine (50 mg/kg; Zoletil, Virbac Laboratory, Carros, France) and xylazine (10 mg/kg; Rompun, Bayer, Mexico), and then 3 μl of PBS or cell suspension (1 × 10^5^) was bilaterally injected into the dentate gyrus of the hippocampus with a Hamilton syringe (26-gauge needle, Hamilton Company, Reno, NV, USA) using a microinfusion pump (KD Scientific, Holliston, MA, USA) in a stereotaxic apparatus. The cell suspension injection rate was 0.5 μl/min. The surgery was performed with an aseptic technique, and antibiotics (gentamicine, 5 mg/kg SC, SHIN POONG. CO. LTD., Seoul, Korea) and pain relief (ketoprofen, Ketoprofen, 5 mg/kg SC, SDC Pharm., Seoul, Korea) were administered to prevent infection before surgery. After surgery, sterilization of the surgical site, antibiotics (gentamicin, 5 mg/kg SC, SHIN POONG. CO. LTD) and analgesics (ketoprofen, 5 mg/kg SC, SDC Pharm) were administered once a day for 3 to 5 days depending on the condition of the animals. For 7 to 10 days after surgery, the condition of the animal was checked at least three times a day by an experienced laboratory animal technician. If abnormal symptoms such as pain reactions were observed, the attending veterinarian was prescribed and treated with additional drugs (tramadol, Tridol, 5 mg/kg IP, Yuhan Co., Seoul, Korea) after treatment. Moreover, a recovery diet was provided to help the animals recover, and fluid therapy (normal saline, 0.1 ml/10 g, IP, Dai Han Pharm. Co. Ltd., Seoul, Korea) was performed as needed.

### Small animal PET/CT imaging

PET/CT imaging was performed as previously reported [27]. Before the scan, the mice were anesthetized with 2% isoflurane in oxygen gas and intravenously injected with 5 to 8% ethanol/saline solution containing 22.2 MBq (0.1 ml of injection volume) of amyloid beta radiotracers (18F-florbetanem; Neuraceq, DuchemBio, Seoul, Korea, http://www.duchembio.com). Images were acquired at 30 min post injection using a NanoScan PET/CT (Mediso Ltd., Budapest, Hungary).

### Immunocytofluorescence staining

The expression of SSEA-3 (1:200, Abcam, Cambridge, U.K., ab16286), CD105 (1:200, BD Bosciences, San Jose, CA, USA, 580839), NeuN (1:200, Merck Millipore, Burlington, MA, USA, ABN78), Nestin (1:500, Santa Cruz Biotechnology Inc., Dallas, Texas, USA, SC-23927), β-III tubulin (1:500, BioLegend, San Diego, CA, USA, 801201), and in cultured hNTSCs was determined by immunofluorescence staining. The cells were fixed with 4% (w/v) PFA and blocked with 1% (w/v) normal goat serum (Jackson ImmunoResearch Laboratories, Inc., West Grove, PA, USA) and then incubated with primary anti-SSEA-3 (1:200, Abcam, ab16286), anti-CD105 (1:200, BD Biosciences, 580839), anti-Nestin (1:500, Santa Cruz Biotechnology Inc., SC-23927), anti-β-III tubulin (1:500, BioLegend, 801201), or anti-NeuN (1:200, Merck Millipore, ABN78) antibodies, and incubated with goat anti-mouse or rabbit Alexa Fluor 488 or 546 antibodies (1:1,000; Molecular Probes, Eugene OR, USA, www.thermofisher.com). The nuclei were labeled with DAPI (1:1000, Sigma-Aldrich), and fluorescence was observed using a Zeiss LSM510 confocal microscope (Carl Zeiss).

### Immunohistofluorescence staining

The obtained human turbinate tissue was fixed with 4% (w/v) PFA and paraffin-embedded sections were processed for staining with p75 NGF receptor (1:500, Abcam, ab3125), Nestin (1:500, Santa Cruz Biotechnology Inc., SC-23927), and β-III tubulin (1:500, BioLegend, 801201). To perform immunofluorescence staining, xylene and ethanol were used to deparaffinize the tissues. The tissues were then washed with PBS, treated with a proteinase K solution for antigen retrieval, blocked with 1% (w/v) normal goat serum (Jackson ImmunoResearch Laboratories, Inc.), and then incubated with primary anti-p75 NGF receptor (1:500, Abcam, ab3125), anti-Nestin (1:500, Santa Cruz Biotechnology Inc., SC-23927), or anti-β-III tubulin (1:500, BioLegend, 801201) antibodies. Then, the tissues were incubated with goat anti-rabbit or anti-mouse Alexa Fluor 488 antibodies (1:1,000; Molecular Probes). The nuclei were labeled with DAPI (1:1000, Sigma-Aldrich), and fluorescence was observed using a Zeiss LSM510 confocal microscope (Carl Zeiss).

For immunohistochemistry, animals were sacrificed at 7 weeks after transplantation. The mice were anesthetized with ketamine (50 mg/kg; Zoletil) and xylazine (10 mg/kg; Rompun) and perfused with 4% paraformaldehyde (Biosesang, Seongnam, Republic of Korea, http://www.biosesang.com/). The excised brain tissue was fixed, embedded, snap frozen in liquid nitrogen, and stored at − 80 °C until use. To investigate Aβ plaques in 5 × FAD mice, brain tissues were cut (14 μm) using a freezing microtome (Leica Camera, Wetzlar, Germany). Tissue sections were pretreated with 97% formic acid for 3 min and then incubated with a mouse anti-beta-amyloid antibody (6E10, 1:100; BioLegend, 803002) for 1 h at RT. Subsequently, the sections were incubated with a biotinylated horse anti-mouse IgG antibody (1:200; Vector Laboratories, Burlingame, CA, USA) and FITC-streptavidin. To detect the immunofluorescence of target molecules, the tissue sections were incubated overnight at 4 °C with the following primary antibodies: anti-Iba-1 (1:500; Wako, Osaka, Japan), anti-NeuN (1:200, Merck Millipore, ABN78), and anti-human nuclear antigen (HuNu, 1:100, Merck Millipore, MAB1281). The nuclei were labeled with DAPI (1:1000, Sigma-Aldrich), and fluorescence was observed using a Zeiss LSM510 confocal microscope (Carl Zeiss).

### Flow cytometry

Single-cell suspensions were prepared from hNTSCs and hBM-MSCs. Cells were incubated for 30 min at 4 °C with stage-specific embryonicantigen-3 (SSEA-3) antibody (1:20, Abcam) followed by Alexa Fluor 488 anti-rat antibody (1:200, Thermo Fisher Scientific). After incubation with the SSEA-3 antibody, the cells were incubated with CD105 antibody (1:50, PE-conjugated, BD Biosciences) for 30 min at 4 °C for double staining. The cells were resuspended in fluorescence-activated cell sorting (FACS) buffer and acquired through FACS Canto II (BD Biosciences) with DIVA software.

### Western blots

Seven weeks after transplantation, half of the brain tissues were homogenized in RIPA buffer (Thermo Fisher Scientific) containing protease inhibitors (GenDEPOT Inc., Barker, TX, USA) and sonicated. The homogenates were centrifuged at 20,000*g* for 20 min at 4 °C, and the supernatant was retained. For Western blot analysis of NEP, BECN1, LC3, and RAB7, protein samples were loaded onto NuPAGE 4–12% (w/v) and 12% (w/v) Bis-Tris Protein Gels (Thermo Fisher Scientific) and transferred to a polyvinylidene difluoride (PVDF) membrane (Roche, Mannheim, Germany). The membrane was blocked with 5% (w/v) milk and incubated with primary anti-NEP (1:1000, Merck Millipore, AB5458), anti-BECN-1 (1:1000, Cell Signaling Technology, Danvers, MA, USA, 3738S), anti-LC3 (1:1000, Cell Signaling Technology, 2775S), anti-RAB7 (1:1000, Proteintech Group Inc., Rosemont, IL, USA,55469-1-AP), and anti-β-actin (1:1000, Santa Cruz Biotechnology, SC47778) antibodies. The membranes were washed and incubated with horseradish peroxidase-conjugated secondary antibodies and developed using enhanced chemiluminescence detection reagents (Thermo Fisher Scientific).

### ELISA assay

Human Aβ40 and Aβ42 and mouse IL-10 and IL-6 were quantified in brain tissue homogenates by ELISA kits according to the manufacturer’s instructions. Aβ40 and Aβ42 ELISA kits (Invitrogen, KHB3481 and KHB3441) and IL-10 and IL-6 ELISA kits (R&D Systems, Inc., Minneapolis, MN, USA, M1000B and M6000B) were used. Each experimental sample was tested in duplicate at least three times.

### Behavioral test

Spatial working memory and learning was assessed by the Morris water maze (MWM) trial performed 6 weeks after cell transplantation. The MWM trial was performed as previously described [28–30] after releasing a nontoxic white colorant into a circular pool (1.5 m in diameter) filled with opaque water (25 ± 1 °C). Testing was performed by an experienced investigator blinded to genotype and treatment group. Prior to the trial, the mice were free swimming for 60 s in the pool to become familiar with the test environment, and then, after platform (10 cm in diameter) was exposed 1 cm above the water surface, the mice were allowed to swim for 60 s, and then placed on the platform for 20 s. The next day, the platform was placed 1 cm below the height of the water surface and the mice were trained to find the hidden in 3 trials per day for 7 days. Each trial was conducted at a different location in one of the quadrants where the platform was not placed and the mice were allowed to search until reaching the platform or until 60 s had lapsed. After the trial, the mice were manually dried with a towel and placed in a warming cage to maintain body temperature. The trial was conducted at a similar time to minimize experimental variables. To evaluate memory retention, a probe trial was conducted on day 8. During the probe trial, the platform was removed from the pool and the mice were released into the pool directly opposite the location of the training target platform and allowed to swim freely for 60 s. Each mouse was automatically monitored, and the time that the mice stayed in each quadrant (zones 1–4) was recorded using the Smart 3.0 Video Tracking System (Panlab, S.L., Barcelona, Spain).

### Quantification and statistical analysis

All data are expressed as the mean (SD) from at least three independent experiments. For the multiple comparison tests, Tukey’s post hoc ANOVA test was used to determine whether group differences were statistically significant. Statistical differences between two different samples were determined with Student’s *t* test. A probability value < 0.05 was considered significant. For quantification of Iba-1- and NeuN-positive cells in brain tissue, cells were counted in four randomly selected nonoverlapping and similar regions per section (five animals per group). For quantification of HuNu-positive cells in brain tissue, cells were counted in three randomly selected nonoverlapping and similar regions per section (four animals per group). Stained cells were counted by using Image-Pro Plus software (Media Cybernetics, Inc., Rockville, MD, USA, http://www.mediacy.com). To analyze amyloid plaque areas, immunofluorescence-positive regions in the brain sections were analyzed by using ZEN imaging software (Carl Zeiss).

## Results

### Characteristics of hNTSCs in culture

**Fig. 1:**
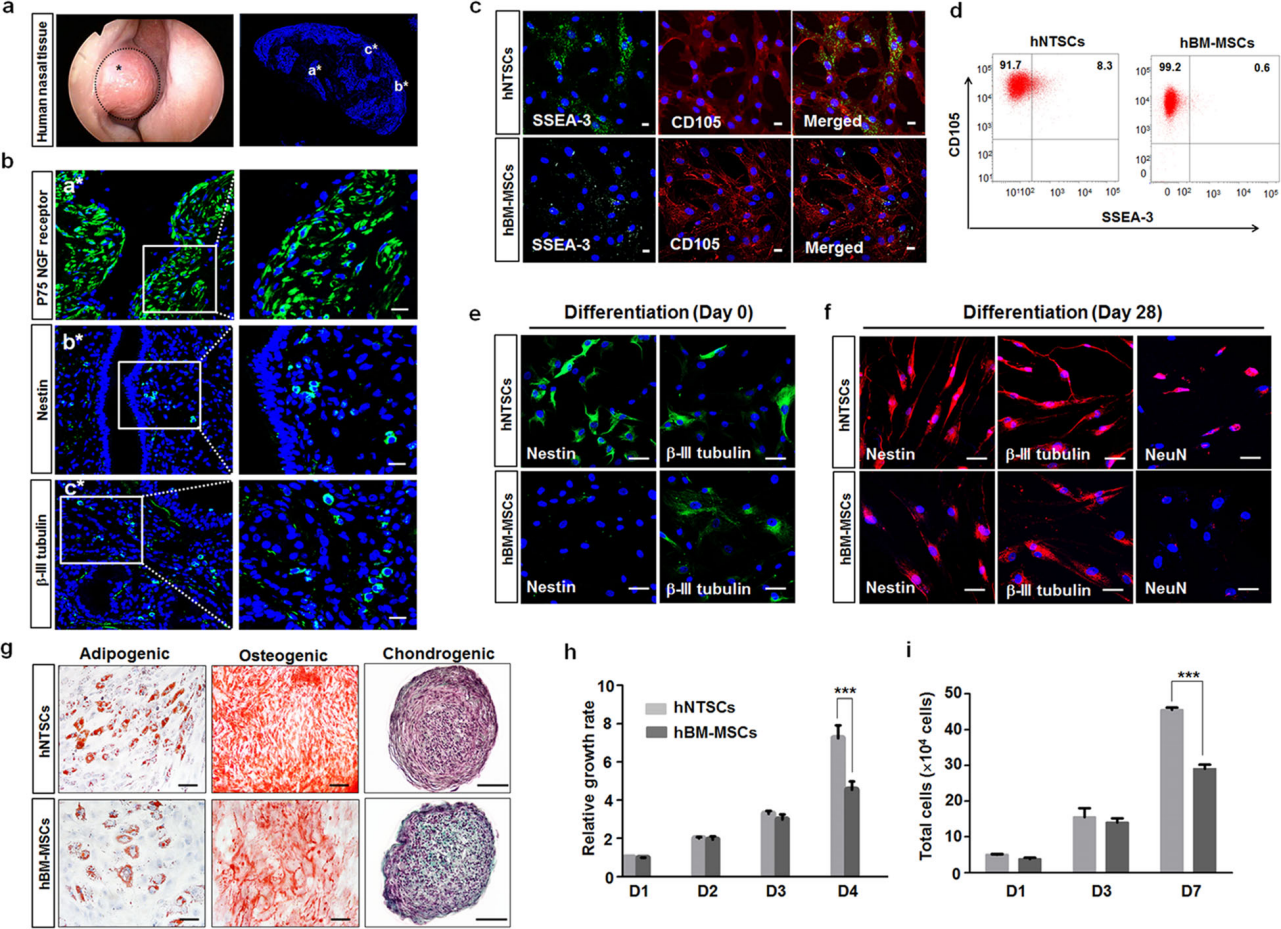
Characterization of hNTSCs. **a** Endoscopic images of the human inferior turbinate in the right nasal cavity and the tissue obtained from turbinectomy stained with DAPI for nuclear labeling. Scale bar: 500 μm. **b** Confocal microscopy images of the human inferior turbinate after staining with antibodies against p75 NGF receptor, nestin, and β-III tubulin (green). Nuclei were labeled with DAPI (blue). Side of the mucosal layer in the inferior turbinate nasal tissue is marked with a* and b*. The submucosal area of the inferior turbinate nasal tissue is marked with c*. Scale bars: 20 μm. **c** Confocal microscopy images of cultured hNTSCs double stained for SSEA3 (green) and CD105 (red) in proliferation medium. Nuclei were labeled with DAPI (blue). Scale bars: 20 μm. **d** Flow cytometry analyses of SSEA-3 and CD105 expression in hNTSCs and hBM-MSCs. **e, f** Confocal microscopy images of hNTSCs and hBM-MSCs stained for nestin and β-III tubulin (green) in culture with proliferation medium and nestin, β-III tubulin, and NeuN (red) after 28 days of incubation in neurogenic differentiation medium. Nuclei were labeled with DAPI (blue). Scale bars: 50 μm. **g** Images of cultured hNTSCs and hBM-MSCs stained with Oil Red O on day 14 of incubation in adipogenic differentiation medium, Alizarin Red S on day 21 of incubation in osteogenic differentiation medium, and Safranin O on day 14 of induction in chondrogenic differentiation medium. Scale bars: 50 μm, 100 μm. **h** Growth rates of hNTSCs and hBM-MSCs incubated in proliferation medium for 4 days after plating. Cell growth was measured using an EZ-Cytox assay kit. Each bar represents relative cell growth (SD). For the multiple comparison tests, two-way ANOVA test was used to determine whether group differences were statistically significant. ***P < 0.001. **i** Cell proliferation of hNTSCs and hBM-MSCs incubated in proliferation medium for 7 days after plating. Cell proliferation was evaluated by trypan blue exclusion assay. Each bar represents viable cell numbers (SD). For the multiple comparison tests, two-way ANOVA was used to determine whether group differences were statistically significant. ***P < 0.001. All images and data are representative of two or three independent experiments

After obtaining inferior turbinate nasal tissue, we investigated whether human inferior turbinate tissue contains neuronal linage cells by staining for neuronal marker or neurotrophin receptor marker. Immunostaining of the inferior nasal tissue showed strong expression of p75 NGF receptor, which is highly expressed in migrating neural crest cells and most sensory neurons during development [31]. Moreover, immunostaining the tissue with anti-Nestin and anti-β-III tubulin antibodies revealed the presence of neural stem cells and immature neurons within the inferior turbinate nasal tissue (Fig. 1a, b). After isolation of hNTSCs from the inferior turbinate nasal tissue, we investigated their potential to give rise to multilineage tissue. Multilineage-differentiating stress-enduring (MUSE) cells have pluripotency to differentiate into all germ layers, similar to embryonic stem cells [32]. These cells are double positive for the expression of CD105 and SSEA-3 [33, 34]. Immunostaining of the hNTSCs with both anti-CD105 and anti–SSEA3 antibodies showed that some hNTSCs were double positive for CD105 and SSEA3, indicating the presence of a MUSE cell population in hNTSCs (Fig. 1c). Cells positive for CD105 but negative for the SSEA-3 marker are not MUSE cells. Moreover, flow cytometry analysis revealed that hNTSCs contained more than 10-fold of CD105-SSEA3 double-positive MUSE cells than hBM-MSCs (Fig. 1d). Under proliferating conditions, hNTSCs strongly expressed the neural stem cell markers Nestin and β-III tubulin, which are differentiating neuron markers (Fig. 1e). hNTSCs cultured in neuronal differentiation medium showed greater expression of the differentiating and mature neuron markers β-III tubulin and NeuN on day 28 of induction. hBM-MSCs showed greater expression of neuronal markers Nestin and β-III tubulin in neuronal differentiation medium, but very weakly expressed mature neuronal marker NeuN (Fig. 1f). During neuronal differentiation, the cytoplasm of hNTSCs was retracted toward the nucleus, followed by enhanced neurite outgrowth. Moreover, hNTSCs and hBM-MSCs cultured in adipogenic differentiation medium, osteogenic differentiation medium, and chondrogenic differentiation medium showed great Oil Red O staining, Alizarin Red S staining, and Safranin O staining on days 14 or 21 of induction (Fig. 1g). The cell growth of cultured hNTSCs was compared with that of hBM-MSCs. hNTSCs showed a much faster growth rate than hBM-MSCs during the 4-day culture (Fig. 1h). Moreover, hNTSCs showed rapid expansion during the 7-day culture, which is important for therapeutic application (Fig. 1i).

### Transplantation of hNTSCs leads to a reduction in Aβ plaque deposition and Aβ levels in 5 × FAD mouse brains

**Fig. 2:**
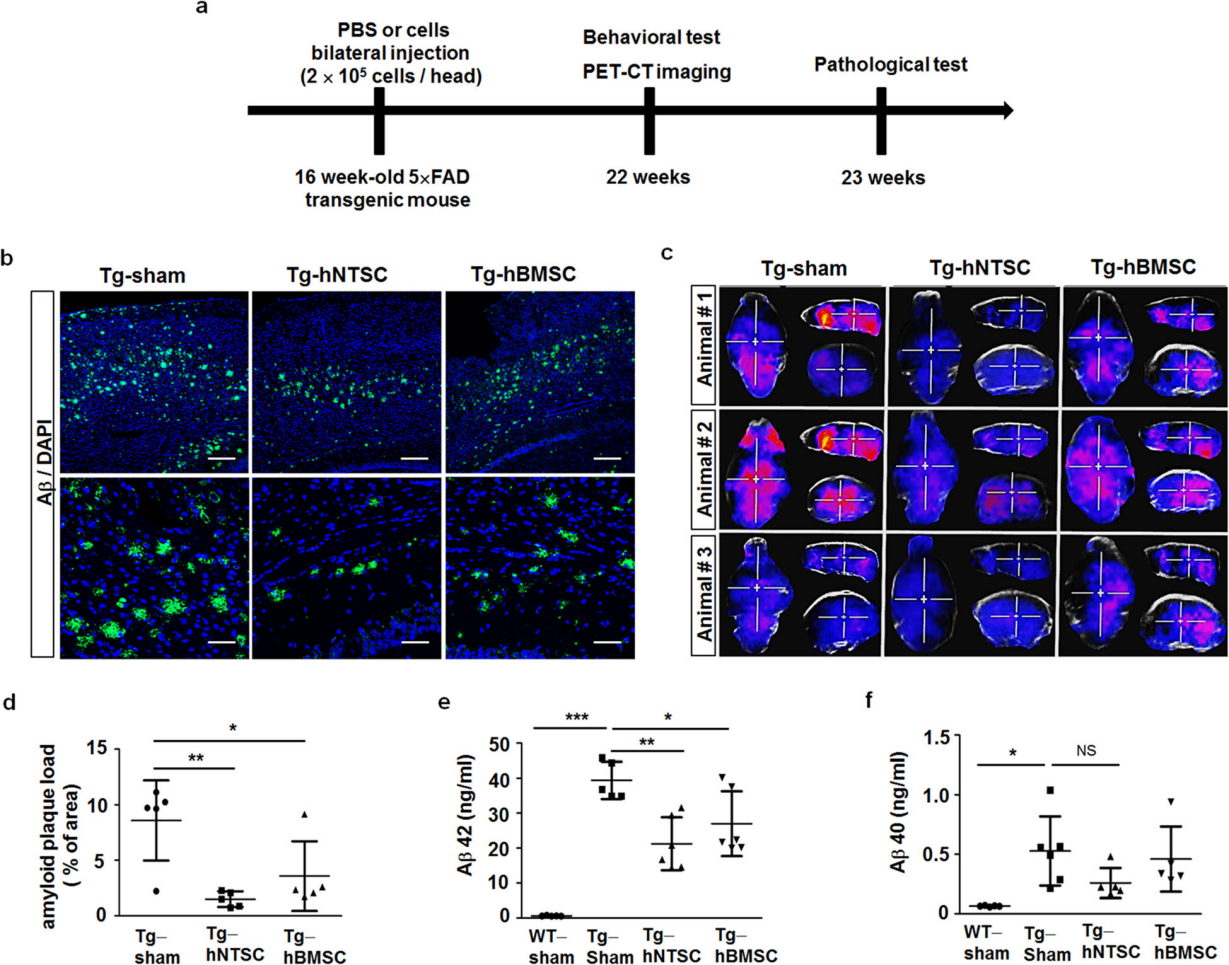
Effects of hNTSC transplantation on Aβ protein load in 5 × FAD transgenic mouse brains. **a** hNTSCs or hBMSCs (1 × 10^5^) were bilaterally injected into the hippocampus of 16-week-old 5 × FAD transgenic mice. AD neuropathological features were analyzed at 6 or 7 weeks after cell transplantation. **b** Confocal microscopy images of Tg-sham, Tg-hNTSC, and Tg-hBMSC brains after staining of OCT-embedded sections with an antibody against 6E10 to detect Aβ deposition (green) at 7 weeks after stem cell transplantation. Nuclei were labeled with DAPI (blue). Scale bars: 100 μm, 50 μm. **c** Sagittal and coronal PET images after [18F] flutemetamol injection in Tg-sham, Tg-hNTSC, or Tg-hBMSC mice at 6 weeks after stem cell transplantation. Increased signals for [18F] flutemetamol retention were detected in Tg-sham or Tg-hBMSC mice compared with Tg-hNTSC mice. **d** The 6E10-positive areas were quantified in the hippocampus and cortex (*n* = 5 per group). Values are the mean (SD). For the nonparametric multiple comparison tests, one-way ANOVA was used to determine whether group differences were statistically significant. *P < 0.05, **P < 0.01. **e, f** Aβ40 and Aβ42 levels in brain tissue lysates of Tg-sham, Tg-hNTSC, and Tg-hBMSC mice were analyzed by a specific Aβ ELISA at 7 weeks after stem cell transplantation (*n* = 5–6 per group). Values are the mean (SD). For the nonparametric multiple comparison tests, one-way ANOVA was used to determine whether group differences were statistically significant. *P < 0.05, **P < 0.01, ***P < 0.001. All images and data are representative of two or three independent experiments

To investigate whether transplantation of hNTSCs modulates AD neuropathological features, we injected PBS, hNTSCs, and hBM-MSCs into the brains of 16-week-old 5 × FAD transgenic mice (Fig. 2a). Aβ plaque deposition in the brain was analyzed by immunostaining and PET-CT at 6 or 7 weeks after injection. Immunostaining of the brain with the Aβ-specific antibody 6E10 visualized that the Aβ plaque load was markedly decreased in both the hippocampal and cortical regions of hNTSC-injected transgenic mice (Tg-hNTSC) compared to transgenic mice injected with PBS (Tg-sham) or hBM-MSCs (Tg-hBMSC) (Fig. 2b). Moreover, a representative comparison of Aβ PET images showed much higher retention of 18F-florbetaben in the brain of Tg-sham mice than in the brains of Tg-hNTSC or Tg-hBMSC mice (Fig. 2c). For quantification of the area occupied by the Aβ plaque in the brain, the immunofluorescence density of 6E10-positive signals was analyzed in the hippocampus and the cortex regions of brain tissue. The average Aβ plaque loads in the brains of Tg-sham, Tg-hNTSC, and Tg-hBMSC mice were 8.6 (3.6%), 1.5 (0.7%), and 3.6 (3.1%), respectively (Fig. 2d). Nonparametric multiple comparison tests of the Aβ plaque load showed significant reductions in Aβ plaque in the hippocampus and cortex of the Tg-hNTSC and Tg-hBMSC groups compared to the Tg-sham group (**P < 0.01, *P < 0.05). Tg-hNTSC mice showed a greater reduction in Aβ plaque load than Tg-hBMSC mice. ELISA of brain homogenates revealed that the levels of soluble Aβ42 were decreased significantly in the Tg-hNTSC and Tg-hBMSC groups compared to the Tg-sham group. Tg-hNTSC mice showed a greater reduction in soluble Aβ42 levels than Tg-hBMSC mice (Fig. 2e). In addition, the levels of soluble Aβ40 were decreased in Tg-hNTSC mice compared to either Tg-sham or Tg-hBMSC mice, but there was no statistically significant difference among the groups (Fig. 2f).

### Transplantation of hNTSCs regulates inflammatory microglial status in 5 × FAD mouse brains

**Fig. 3:**
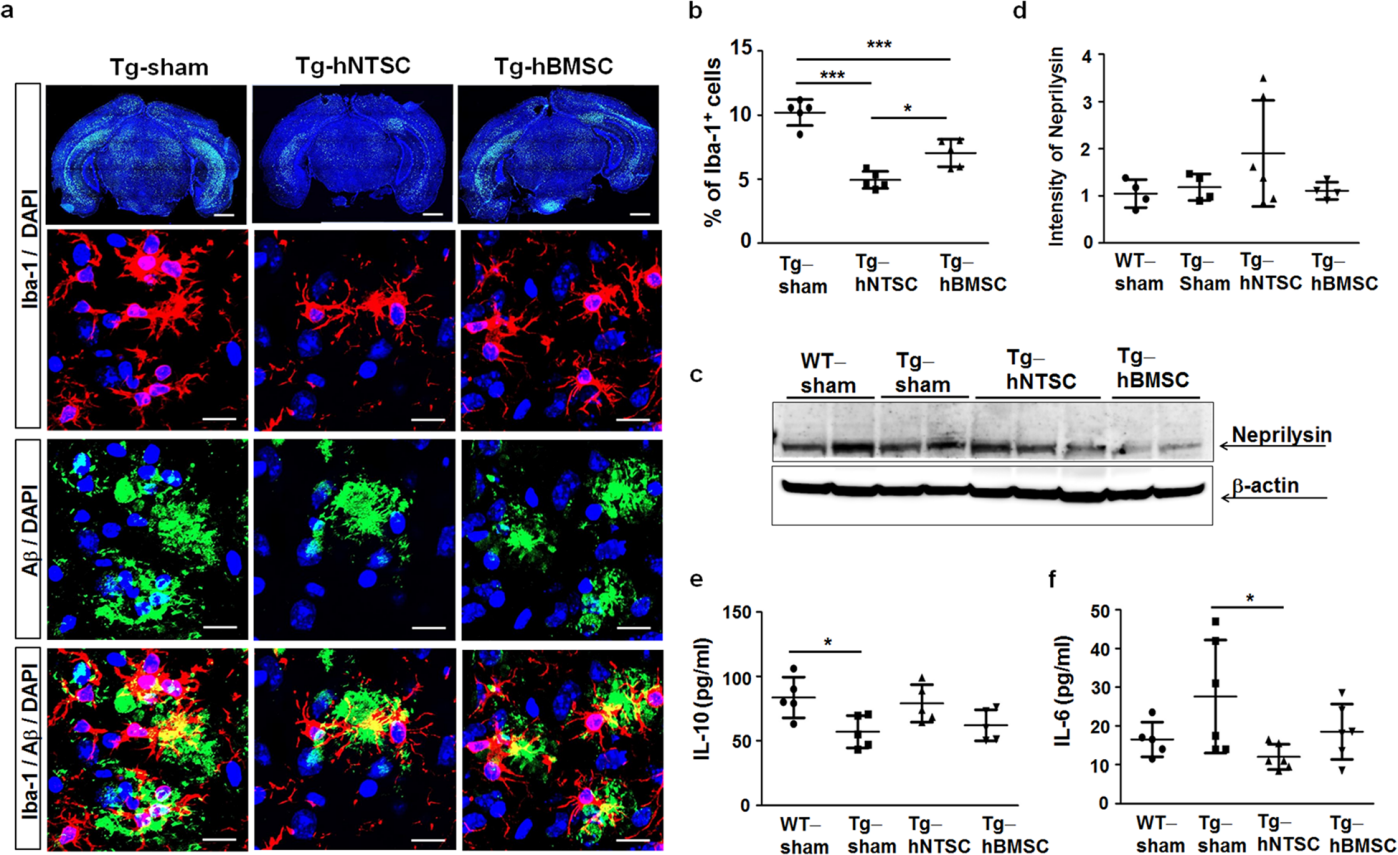
Effects of hNTSC transplantation on the number of inflammatory microglia in 5 × FAD mouse brains. **a** Confocal microscopy images of the brains in Tg-sham, Tg-hNTSC, and Tg-hBMSC mice after double staining of OCT-embedded sections with antibodies for Iba-1 to detect microglia (green, red) and 6E10 (green) at 7 weeks after stem cell transplantation. Clustered microglial cells were detected in the vicinity of Aβ plaques in the brain tissue. Nuclei were labeled with DAPI (blue). Scale bars: 1000 μm, 20 μm. **b** Iba-1-positive cells were counted in the hippocampus and cortex (*n* = 5 per group). Values are the mean (SD). For the nonparametric multiple comparison tests, one-way ANOVA was used to determine whether group differences were statistically significant. *P < 0.05, *** *P* < 0.001. **c** Western blots of SDS-PAGE gels of proteins extracted from the brain tissues of WT-sham, Tg-sham, Tg-hNTSC, and Tg-hBMSC mice using a primary anti-NEP antibody at 7 weeks after stem cell transplantation. β-actin was used as a loading control. **d** Values are the mean (SD). **e, f** The levels of the anti-inflammatory cytokines IL-10 and IL-6 proinflammatory cytokines in the brain tissue lysates of Tg-sham, Tg-hNTSC, and Tg-hBMSC mice were analyzed by specific ELISA at 7 weeks after stem cell transplantation (*n* = 5–6 per group). Values are the mean (SD). For the nonparametric multiple comparison tests, one-way ANOVA was used to determine whether group differences were statistically significant. *P < 0.05. All images and data are representative of three independent experiments

Microglia that are activated and concentrated around Aβ plaques are a feature of the AD brain [35, 36]. Activated microglia can damage neuron and synapse function and secrete inflammatory cytokines that can injure neurons directly and cross the blood brain barrier, initiating systemic inflammation [37–39]. Brain inflammation is a complicated process in which resident microglia, neutrophils, monocytes, astrocytes, and neurons all play a role. Iba-1 is expressed in both microglia and monocytes [40, 41], so Iba-1 positive cells could be peripheral macrophages/monocytes homing to areas of brain damage. Iba-1 is nevertheless a good marker for cell-based inflammatory reactions in the brain. To investigate whether transplantation of hNTSCs mediates microglial status, PBS, hNTSCs, and hBMSCs were injected into the brains of 5 × FAD transgenic mice. At 7 weeks after transplantation, microglial numbers were analyzed by immunofluorescence staining for the microglial marker Iba-1. Iba-1 positive cells were counted in the hippocampus and the cortex regions of brain tissue and data was presented as the mean percentage of positive cells. The average percentages of Iba-1 positive cells in brain sections of Tg-sham, Tg-hNTSC, and Tg-hBMSC mice were 10.2 (1.0%), 4.9 (0.7%), and 7.0 (1.1%), respectively (Fig. 3a, b). Confocal microscopy images and quantitative analysis showed that transplantation of hNTSCs significantly reduced the expression of inflammatory microglia in the hippocampal and cortical regions of Tg-hNTSC mice compared to those of Tg-sham mice (***P < 0.001). The reduction in microglial numbers was much greater in Tg-hNTSC mice than in Tg-hBMSC mice. Moreover, double immunofluorescence staining for the microglial markers Iba-1 and 6E10 for Aβ deposition demonstrated that microglial cells were clustered around the Aβ plaque load, and the amounts of both Aβ plaque and microglial cells were reduced remarkably in Tg-hNTSC mice compared with Tg-sham mice.

We next investigated the expression levels of neprilysin (NEP), which is an important regulator of cerebral Aβ levels, by proteolytic degradation of Aβ [42]. At 7 weeks after hNTSC transplantation, the levels of NEP were approximately 2.0-fold higher in Tg-hNTSC mice than in Tg-sham or Tg-hBMSC mice, but there was no significant difference in NEP levels between Tg-hNTSC and Tg-sham mice due to variations among individual mice (Fig. 3c, d). Moreover, we investigated the effect of transplantation of hNTSCs on the levels of inflammatory cytokines in the brains of 5 × FAD transgenic mice. ELISA of brain homogenates showed that the level of IL-10, an anti-inflammatory cytokine, was increased significantly in Tg-sham mice compared with wild-type mice of the same age that were injected with PBS (WT-sham), but there were comparable levels of IL-10 in Tg-hNTSC and Tg-sham mice (Fig. 3e). In WT-sham mice, the secretion level of IL-6, a proinflammatory cytokine, was increased compared with that in Tg-sham mice, and this level was significantly decreased in Tg-hNTSC mice but not in Tg-hBMSC mice (Fig. 3f). These data suggest that transplantation of hNTSCs may modulate microglial activity, which leads to a reduction in microglial numbers and regulation of proinflammatory and anti-inflammatory cytokine levels in 5 × FAD transgenic mice.

### Transplantation of hNTSCs upregulates autophagic capacity in the brains of 5 × FAD

**Fig. 4:**
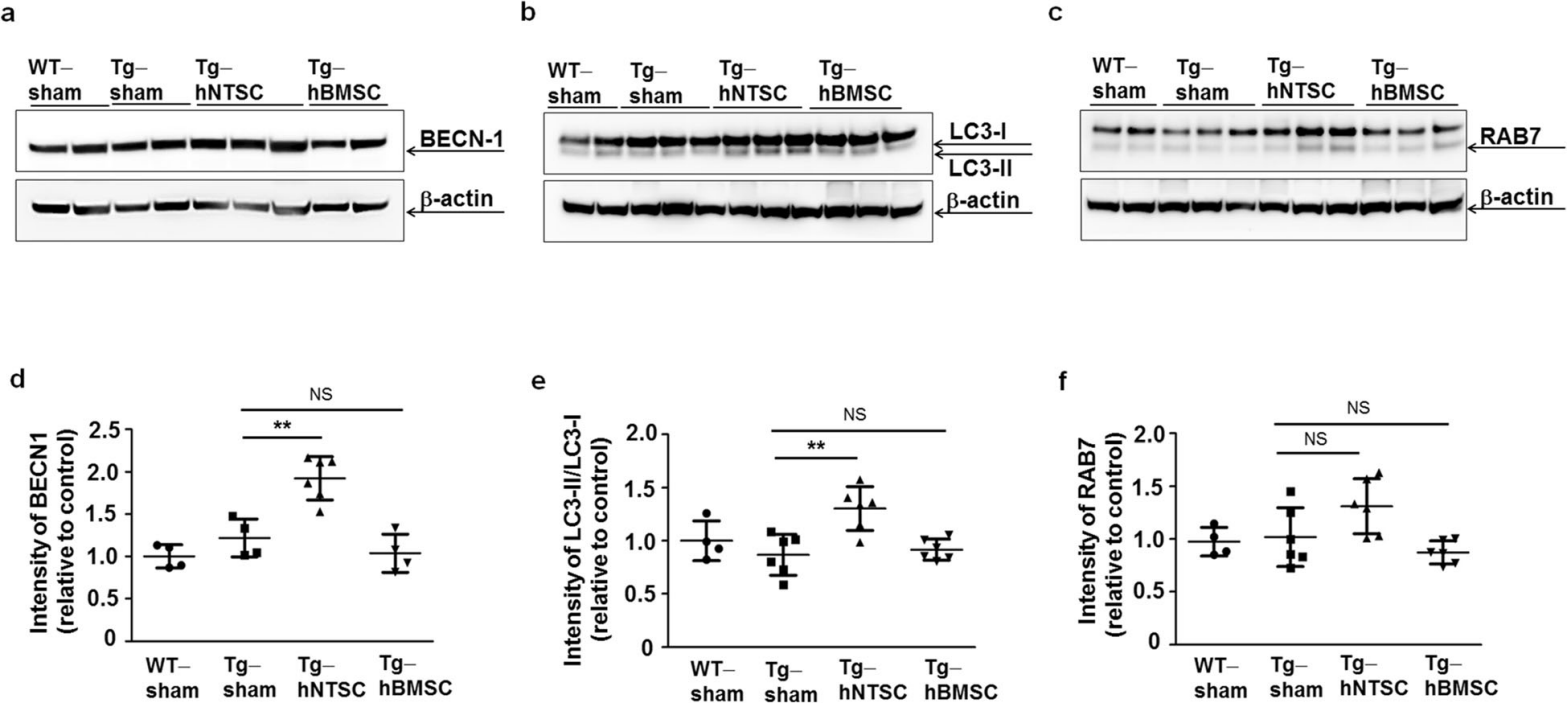
Effects of hNTSC transplantation on autophagy modulation in 5 × FAD mouse brains. **a–c** Western blots of SDS-PAGE gels of brain tissue extracts of WT-sham, Tg-sham, Tg-hNTSC, and Tg-hBMSC mice using primary anti-BECN1, anti-LC3, and anti-RAB7 antibodies at 7 weeks after stem cell transplantation. β-actin was used as a loading control. **d, f** Values are the mean (SD). For the nonparametric multiple comparison tests, one-way ANOVA was used to determine whether group differences were statistically significant. **P < 0.01. **e** Values are the mean (SD). For the nonparametric multiple comparison tests, one-way ANOVA was used to determine whether group differences were statistically significant. **P < 0.01. All data are representative of three independent experiments

Autophagy plays a neuroprotective role in various neurodegenerative diseases [43, 44]. In particular, stimulation of autophagy increases therapeutic benefits against Aβ-induced toxicity in AD animal models [45, 46]. To investigate whether transplantation of hNTSCs regulates autophagic capacity in 5 × FAD transgenic mice, we subjected SDS-PAGE gels of brain extracts from Tg-sham, Tg-hNTSC, or Tg-hBMSC mice to Western blotting analysis. BECN1, a key regulator that determines autophagic capacity and promotes cell survival [47], was expressed at significantly higher levels in Tg-hNTSC mice than in Tg-sham mice, but there were comparable levels of BECN1 in Tg-hBMSC and Tg-sham mice (Fig. 4a, d). Moreover, we further examined the expression of LC3 and RAB7 in5 × FAD transgenic mice injected with PBS, hNTSCs, or hBMSCs. LC3 is a key protein in the autophagy pathway and the most widely used marker for monitoring autophagy. The conversion of LC3I to LC3-II reflects autophagy and autophagy-related processes, including autophagic cell death [48, 49]. The LC3-II/LC3-I ratio was significantly increased in 5 × FAD transgenic mice compared with PBS-injected mice (Fig. 4b, e). However, there were no significant differences between Tg-hBMSC and Tg-sham mice. Moreover, Tg-hNTSC mice showed higher levels of RAB7, which is required for the final maturation of late autophagic vacuoles, than Tg-sham or Tg-hBMSC mice (Fig. 4c, f), suggesting that hNTSCs could reduce Aβ levels in the brain through modulation of autophagic capacity.

### Transplantation of hNTSCs improves the cognitive impairment of 5 × FAD mice

**Fig. 5:**
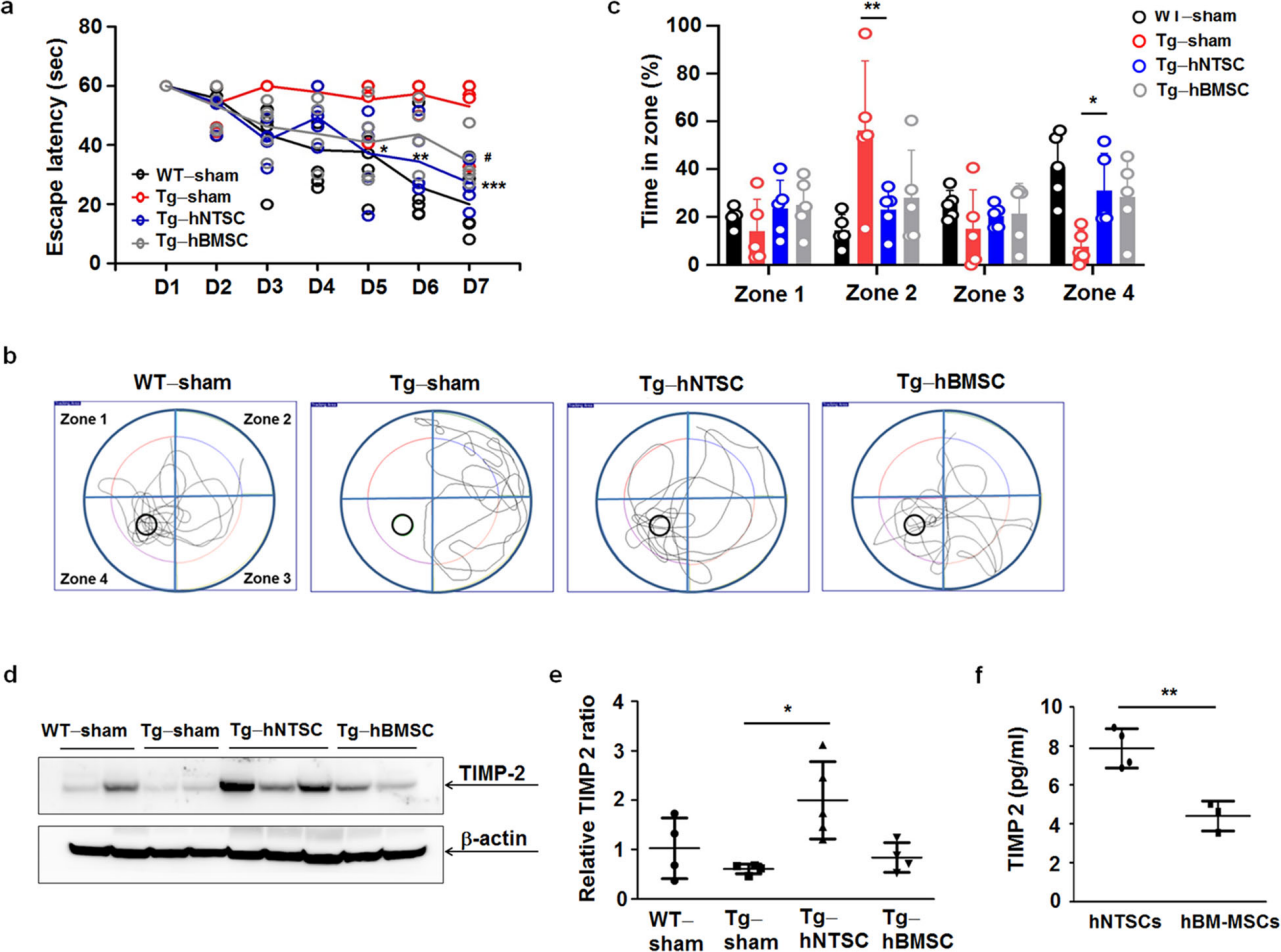
Effects of hNTSC transplantation on cognitive deficit in 5 × FAD mouse brains. **a** The escape latency was the time for each group to find a hidden platform within 60 s. For the multiple comparison tests, two-way ANOVA was used to determine whether group differences were statistically significant. ^#^*P* < 0.05, *P < 0.05, **P < 0.01, ***P < 0.001. **b, c** Probe test images of whether each group remembered the platform location in zone 4 during training. Data were expressed as the percentage (%) of time spent in each zone within 60 s. For the multiple comparison tests, two-way ANOVA test was used to determine whether group differences were statistically significant. *P < 0.05, **P < 0.01, ***P < 0.001. **d** Western blots of SDS-PAGE gels of brain tissue extracts of WT-sham, Tg-sham, Tg-hNTSC, and Tg-hBMSC mice using a primary anti-TIMP2 antibody at 7 weeks after stem cell transplantation. β-actin was used as a loading control. **e** Values are the mean (SD). For the nonparametric multiple comparison tests, one-way ANOVA was used to determine whether group differences were statistically significant. **f** TIMP-2 levels of hNTSCs and hBM-MSCs in culture, analyzed by TIMP-2 ELISA. Values are the mean (SD). Statistical differences between two different samples were determined with Student’s *t* test. **P < 0.01. All data are representative of two or three independent experiments

To investigate the potential of hNTSCs for protecting learning and memory ability, we performed Morris water maze training for 5 × FAD transgenic mice at 6 weeks after transplantation. For spatial learning memory test, 60 s learning trials with 1.5-h intervals allowed mice to learn the location of target platform. During the 7-day training period, WT-sham, Tg-hNTSC, and Tg-hBMSC mice showed a progressive improvement in the ability to find the platform in a time-dependent manner. However, Tg-sham mice showed significant cognitive impairment, and there were no differences in escape latency at day 7 compared to day 1 of the training period (Fig. 5a). The average escape latencies of WT-sham, Tg-sham, Tg-hNTSC, and Tg-hBMSC mice were 20.0 (11.8 s), 53.1 (11.6 s), 27.3 (7.9 s), and 34.4 (8.0 s), respectively. Compared with Tg-sham mice, Tg-hNTSC mice showed significant improvement on days 5, 6, and 7 of the training period (*P < 0.05, **P < 0.01, ***P < 0.001), but Tg-hBMSC mice showed significant improvement on day 7 of the training period (^#^*P* < 0.05). Moreover, the probe trial showed that the WT-sham and Tg-hNTSC mice spent significantly more time in the target quadrant zone 4 when compared with the Tg-sham group (*P < 0.05, **P < 0.01). The average preference for the target quadrant of WT-sham, Tg-sham, Tg-hNTSC, and Tg-hBMSC was 41.0 (14.1%), 7.6 (6.8%), 31.0 (15.6%), and 28.3 (15.7%), respectively (Fig. 5b, c). These results demonstrated that transplantation of hNTSC significantly enhanced performance in the Morris water maze. However, there was no significant difference in preference for the target quadrant between Tg-sham and Tg-hBMSC mice.

Tissue inhibitor of metalloproteinases 2 (TIMP2) is associated with various neuropathological conditions and has been demonstrated to reduce TIMP2 levels in the plasma of patients with frontotemporal dementia [50, 51]. Castellano and colleagues have shown that systemic treatment with TIMP2 improves hippocampus-dependent cognition in aged mice [52]. Here, we investigated whether hNTSC transplantation could regulate the levels of TIMP2 in 5 × FAD transgenic mice. Western blots of SDS-PAGE gels showed that TIMP2 levels were, on average, 5-fold higher in Tg-hNTSC mice than in Tg-sham mice at 7 weeks after transplantation (Fig. 5d, e), but there was no significant increase in TIMP2 levels between Tg-sham and Tg-hBMSC mice. In addition, approximately 2-fold higher TIMP2 levels were observed in cultured hNTSCs than in hBMSCs (Fig. 5f), supporting the conclusion that there are much higher TIMP2 levels in Tg-hNTSC mice than in Tg-hBMSC mice, as shown in Fig. 5d, e. These data support the greater cognitive improvement observed in Tg-hNTSC mice than in Tg-sham or Tg-hBMSC mice, as shown in Fig. 5a–c.

### Transplantation of hNTSCs increases neuronal survival in the brains of 5 × FAD mice

**Fig. 6:**
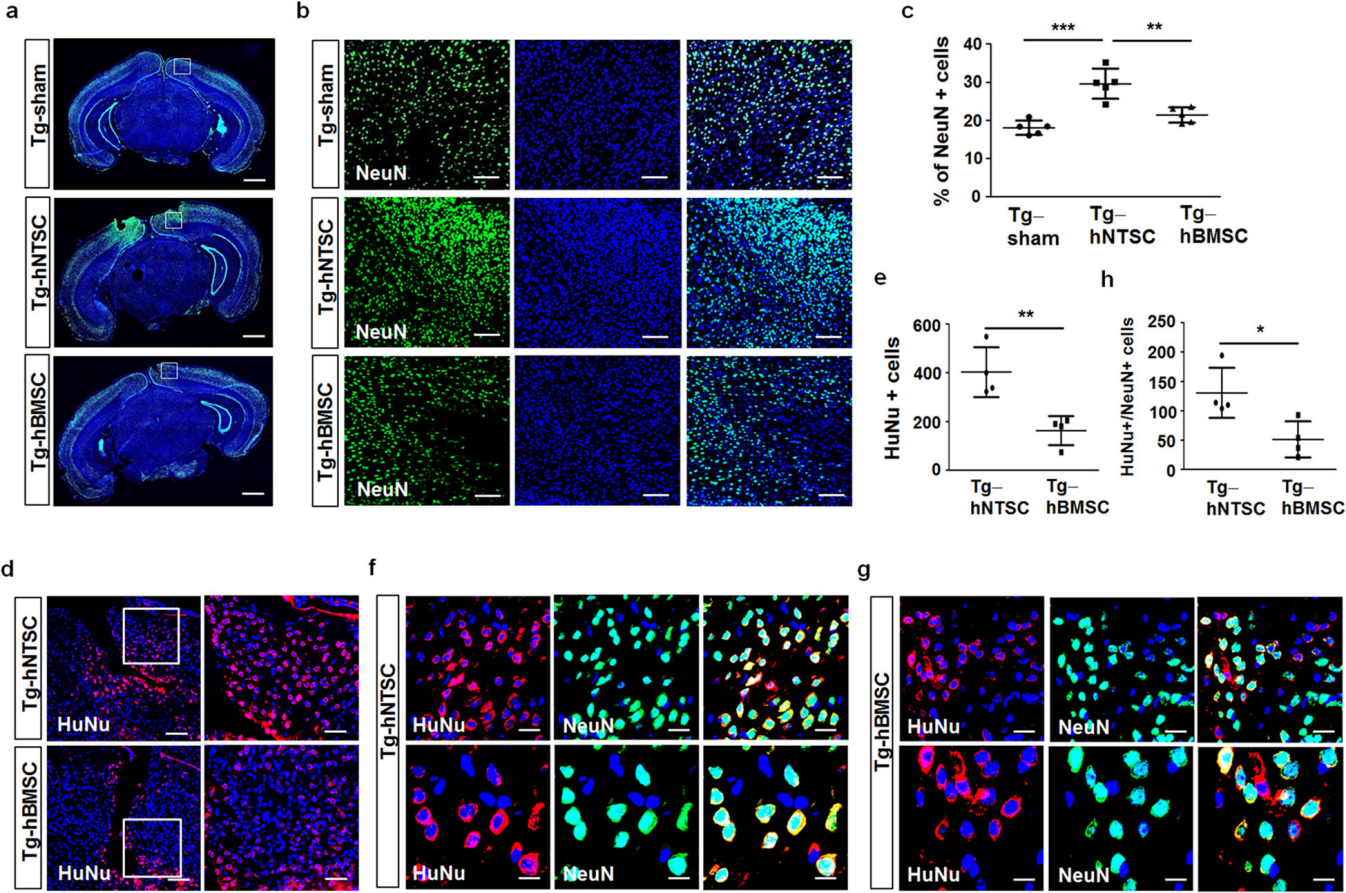
Effects of hNTSC transplantation on neuronal survival and the engraftment of hNTSCs in 5 × FAD mouse brains. **a, b** Confocal microscopy images of the brains of Tg-sham, Tg-hNTSC, and Tg-hBMSC mice after staining of brain tissue sections with an antibody against NeuN to detect neurons (green) at 7 weeks after stem cell transplantation. Nuclei were labeled with DAPI (blue). Scale bars: 1000 μm, 100 μm. **c** NeuN-positive cells were counted in the cortex (*n* = 5 per group). Values are the mean (SD). For the nonparametric multiple comparison tests, one-way ANOVA was used to determine whether group differences were statistically significant. **P < 0.01, ***P < 0.001. **d** Confocal microscopy images of the brains of Tg-hNTSC and Tg-hBMSC mice after staining of brain tissue sections with an antibody against HuNu to detect engrafted human cells (red) at 7 weeks after stem cell transplantation. Nuclei were labeled with DAPI (blue). Scale bars: 50 μm, 20 μm. **e** HuNu-positive cells were counted around the stereotaxic injection site in the brains (*n* = 4 per group). Values are the mean (SD). Statistical differences between two different samples were determined with Student’s *t* test. **P < 0.01. **f, g** Confocal microscopy images of the brains of Tg-hNTSC and Tg-hBMSC mice after double staining of brain tissue sections with antibodies against HuNu (red) and NeuN (green) at 7 weeks after stem cell transplantation. Nuclei were labeled with DAPI (blue). Scale bars: 20 μm, 10 μm. **h** HuNu-NeuN double-positive cells were counted in the cortex (*n* = 4 per group). Values are the mean (SD). Statistical differences between two different samples were determined with Student’s *t* test. *P < 0.05

To investigate whether transplantation of hNTSCs protects against neuronal cell death induced by toxic amyloid plaque, PBS, hNTSCs, and hBMSCs were injected into the brains of 5 × FAD transgenic mice. At 7 weeks after transplantation, histological sections were analyzed by immunofluorescence staining for the NeuN neuronal marker. NeuN-positive cells were counted in the cortical regions of brain tissue and data was presented as the mean percentage of positive cells. The average percentages of NeuN-positive cells in brain sections of Tg-sham, Tg-hNTSC, and Tg-hBMSC mice was 18.1 (1.9%), 29.2 (3.9%), and 21.4 (2.0%), respectively (Fig. 6a–c). Confocal microscopy images and quantitative analysis showed that transplantation of hNTSCs significantly increased the number of NeuN-positive cells in cortical regions of 5 × FAD transgenic mice compared with that in Tg-sham mice or Tg-hBMSC (***P < 0.001, **P < 0.01), suggesting that hNTSCs inhibit progressive neuronal loss in AD. However, the number of neurons in the brain in Tg-hBMSC mice was comparable to that in the brain in Tg-sham mice. Moreover, the transplanted human cells and their differentiation status were analyzed by double immunofluorescence staining for HuNu and NeuN. At 7 weeks after transplantation, approximately 2.5-fold more human cells were detected around the stereotaxic injection site in the brains of Tg-hNTSC mice than in the brains of Tg-hBMSC mice (Fig. 6d, e). Moreover, approximately 3.0-fold more cells in Tg-hNTSC mice were double positive for HuNu and NeuN than in those of Tg-hBMSC (Fig. 6f–h), suggesting that more human cells engrafted into the brain differentiated to express neuron protein in Tg-hNTSC mice.

## Discussion

There have been an increasing number of stem cell trials in patients with AD [9, 53]. The main concept of stem cell therapy is the capacity of transplanted stem cells to differentiate into neuronal cells to replace damaged neurons and to stimulate the endogenous neuronal repair system by secreting neurotrophic factors or neuroprotective cytokines that are crucial for therapy [3–5]. There is much evidence for the efficacy of MSC therapies in animal models of AD. The transplantation of human MSCs improves cognitive functions and alleviates AD neuropathological symptoms [6–8]. In the first clinical trial that used stem cells to treat AD, Kim et al. evaluated the effect of allogenic human UCB-MSC transplantation in patients with AD in a phase I clinical trial [9]. Although the therapeutic effects on cognition and AD pathology were not confirmed, no safety issues were observed in the patients at 24 months after transplantation. A number of stem cell-based clinical trials including hUCB-MSCs, hBM-MSCs, and placenta-derived MSCs for the treatment of AD are currently in progress, and some will begin in the future [9, 53].

Preclinical stem cell studies have demonstrated that stem cells are a promising therapeutic agent, but significant design differences between preclinical and clinical trials may affect clinical translation. To ensure the therapeutic promise of stem cells in AD patients, there are some issues to be evaluated prior to a clinical trial. It is important to identify the properties of the stem cells to be used, such as cell immunogenicity, cryopreservation, and cell types, and optimize the experimental conditions to reflect the clinical reality, such as animal models for evaluation, the time for transplantation, and delivery routes to advance clinical translation.

Nearly 95% of patients with AD are classified as having sporadic AD, which is caused by genetic factors and environmental risk factors [54]. However, a number of drug candidates show therapeutic effects in preclinical models of familial AD. There are common pathologies between familial AD and sporadic AD, such as the deposition of Aβ, the hyperphosphorylation of Tau, and neuronal loss, but their differences cause failure in the development of therapeutic drugs [55]. Recently, many studies have focused on ideal AD animal models, including natural aging-based AD-like canine models, to overcome the limitations of treatment and predict validity in human clinical applications [56, 57]. Aged dogs offer an advantage over nontransgenic aged rodent species because they naturally produce Aβ, which has an identical amino acid sequence to the human protein and results from similar biochemical processing of the amyloid precursor protein. Aged beagles have been shown to develop cognitive dysfunction associated with neuropathology that resembles certain aspects of human AD-related cognitive deficits [57]. Moreover, guinea pigs are a developing animal model of AD featuring a human-like Aβ sequence with age-dependent diffuse accumulation of amyloid pathology [58]. In the treatment of AD using stem cells, efficacy evaluation using an animal model that has high similarity to the patient population likely to be treated in clinical trials will be an important issue.

NCSCs represent an adult dormant stem cell population that is found in various adult tissues, including the hard palate, oral mucosa, periodontal ligament, and nasal inferior turbinate [13–20]. NCSCs have been shown to have the capacity to give rise to diverse mesenchymal phenotypes, adipocytes, and chondrocytes under specific conditions in vitro and in vivo [59–61]. Notably, hNCSCs could be advantageous for treatment of neurodegenerative disorders because of their high potency for differentiation into the ectodermal lineage [15, 62, 63]. Recently, it has been reported that human epidermal NCSCs successfully differentiated into functional dopaminergic (DA) neurons in in vitro culture [64]. Moreover, transplanted hNCSCs have been shown to differentiate into DA neurons in the brain and improve functional recovery in rat models of Parkinson’s disease [65].

hNTSCs can be easily isolated from inferior turbinate tissue removed during turbinate resection via minimally invasive collection procedures. hNTSCs have shown a strong ability to proliferate and differentiate into multiple cell types in culture. Moreover, hNTSCs have demonstrated preservation of multiple biological characteristics of stem cells regardless of donor age or passage number [22, 25]. It is important to preserve and store viable biological samples frozen over long periods of time in cryopreservation [66]. Immediately after thawing, cell viability varied from approximately 50 to 100% [67, 68]. In many immunotherapy trials, MSCs have been administered within a few hours after thawing of cryopreserved cells. This would be feasible if the thawed cells preserved their viability, safety, and multipotency [69]. hNTSCs showed a viability of more than 90% immediately after thawing, suggesting that the cell characteristics of hNTSCs are well maintained during long-term cryopreservation, which can be an advantage for treatment with hNTSCs. Further research on the cell multipotency and therapeutic effects of hNTSCs immediately after thawing will be needed.

Immunosuppressive treatments are necessary to prevent immune-borne transplant damage or rejection [70]. These interventions are of particular importance given the increasing need for solid organ and tissue replacement using stem cells. MSCs exhibit immunomodulatory properties, which increases their therapeutic applicability for the treatment of degenerative and inflammatory diseases. The therapeutic mechanism of MSCs is multifaceted, but they are generally thought to enable damaged tissues to form a balanced inflammatory and regenerative microenvironment [71, 72]. Human-induced pluripotent stem cell-derived neural crest stem cells have been shown to have a nonimmunogenic molecular phenotype and have the potential for inflammatory immune cell suppression by inhibiting T cell activation (cell proliferation and production of inflammatory cytokines) in vitro [73]. Recently, hNTSCs were found to exhibit a nonimmunogenic phenotype and to secreted a number of cytokines and chemokines, including IL-4, IL-6, IL-8, IL-10, IP-10 (CXCL10), and RANTES (CCL5), that are known to be involved in immunomodulation through Toll-like receptor (TLR) 4 stimulation, suggesting that TLR4 is related to the immune-modulating functions of hNTSCs [74]. Further investigations of the immunomodulatory properties of hNTSCs are needed to exploit the improvement of hNTSC-based therapeutic strategies.

Extending these promising findings, we validated the feasibility of hNTSCs as a potent alternative source of stem cells for translation applications in treating AD. Prior to investigating the therapeutic potential of hNTSCs in a transgenic mouse model of AD, hNTSCs were shown to contain more than 10-fold of MUSE cell population than hBM-MSCs (Fig. 1c, d). MUSE cell is defined as a population of cells with the potency to differentiate into three germ layers from a single cell [32]. MUSE cells comprise a very small population of MSCs and are double positive for expression of the MSC marker CD105 and the pluripotent embryonic stem cell marker SSEA3 [32–34]. MSCs comprise nontumorigenic and multipotent heterogeneous cell populations [33]. However, MSCs exhibit a low degree of triploblastic differentiation into three germ layers, namely, the ectoderm, mesoderm, and endoderm [11, 12, 32], which limits the use of MSCs in regenerative medicine. It is possible that the low potency of triploblastic differentiation in MSCs is due to the small percentage of MUSE cells present in the MSC population [32, 33, 75].

Neural crest cells originate in the ectoderm at the margins of the neural tube, and during the process of development, neural crest cells migrate out from their niche between the newly formed ectoderm and the neural tube. After that, they give rise to mesodermal cell types as well as ectodermal cell types [76, 77]. NCSCs are not only present in the embryonic neural crest, but also in various neural crest-derived tissues in the fetal and even adult organism. Recently, many reports have demonstrated that NCSCs behave as multipotent self-renewing stem cells/progenitors [76], showing the ability to differentiate into multiple lineages under the appropriate conditions in vitro and in vivo [77, 78]. In light of these characteristics, highly multipotent adult cells derived from the embryonic neural crest represent an extremely important stem cell population [79] with a differentiation potential that is surpassed only by that of pluripotent embryonic stem cells. Indeed, sphere-forming adult NCSCs seem to harbor a fascinatingly broad differentiation potential, especially in regard to the generation of neuronal and glial cells, osteogenic cells, adipocytes, and chondrocytes [15, 80–82]. hNTSCs exhibited expression of neural proteins, including the neural stem cell marker Nestin and the differentiating neuron marker β-III tubulin , under proliferation (Fig. 1e) and differentiation conditions in vitro (Fig. 1f). In addition, hNTSCs showed the capacity to give rise to diverse mesenchymal phenotypes, including adipocytes, osteocytes, and chondrocytes under specific conditions in vitro (Fig. 1g). Moreover, hNTSCs showed greater growth and expansion in culture (Fig. 1h, i), which is important for therapeutic application of hNTSCs. These findings suggest that hNTSCs may be advantageous for use treating AD in terms of their great neural properties and cell growth.

A major issue of stem cell therapy in AD is the reduction of AD neuropathological features in the brain. To evaluate the therapeutic potential of hNTSCs for AD pathology in vivo, we directly injected cells into the brains of 5 × FAD mice that overexpress five familial AD transgenes. In vivo neuroimaging revealed greatly decreased plaque deposition in the brains of Tg-hNTSC mice compared with those of Tg-sham mice, while little decrease was observed in Tg-hBMSC mice, although there were differences among individuals. The levels of soluble Aβ40 and Aβ42 were also decreased significantly in Tg-hNTSC mice (Fig. 2). The decreased number of Aβ plaques in the AD brain was consistent with a significant improvement in cognitive function in Tg-hNTSC mice compared with Tg-sham mice (Fig. 5).

A number of studies have demonstrated the Aβ plaques and an inflammatory response are main features of AD pathogenesis [83–85]. Inflammation in the brain has different functions; it plays a neuroprotective role in the acute phase but a detrimental role during the chronic phase. Chronic inflammation in the brain causes microglial activation, which damages neuron and synapse function and leads to secretion of inflammatory cytokines that can injure neurons directly and cross the blood brain barrier, initiating systemic inflammation [37–39, 86–89]. It has been reported that transplantation of amniotic MSCs into human neural stem cells increased microglia in the mouse AD brain; microglia promote Aβ clearance and may inhibit neurodegeneration [30]. In other studies, the number of Iba-1-positive microglia was increased at 1 week but decreased at 12 weeks after transplantation of amniotic MSCs. Moreover, the microglial density was decreased significantly at 12 weeks after cell transplantation [90]. Another report showed that NTSCs decreased the number of microglia in the AD mouse brain at 2.5 months after transplantation [91]. Interestingly, we found that the numbers of microglia were decreased significantly in the hippocampal and cortical regions of Tg-hNTSC mice compared with those of Tg-sham mice at 6 or 7 weeks after transplantation, and microglia were observed around Aβ plaques (Fig. 3a, b). Moreover, we found that the levels of secreted cytokines were changed at 7 weeks after transplantation; the level of the proinflammatory cytokine IL-6 was decreased significantly, whereas the level of the anti-inflammatory cytokine IL-10 was increased, although there was no significant difference in IL-10 levels between Tg-sham and Tg-hNTSC mice (Fig. 3e, f). These data suggest that transplantation of hNTSCs could modulate the number of microglia and the level of inflammatory cytokines to restore the immune status of the AD brain.

In the present study, we further investigated the therapeutic effect of hNTSCs in terms of reducing Aβ deposition via autophagic capacity in the AD mouse brain. There is evidence implicating Aβ plaques in modulation of autophagy. Autophagy is essential for the clearance of detrimental Aβ aggregates and thus plays a critical role in maintaining Aβ homeostasis in the AD-related microenvironment [28, 92, 93]. There are several lines of evidence that small molecular compounds or stem cells that can activate autophagy or lysosomal proteolysis can also markedly decrease the Aβ load in AD [45, 46]. Tg-hNTSC mice showed significant upregulation of BECN1, a key determinant in the regulation of autophagic capacity, compared with that seen in Tg-sham or Tg-hBMSC mice at 7 weeks after transplantation (Fig. 4a). Moreover, transplantation of hNTSCs increased the LC-II/LC3-I ratio in AD mice (Fig. 4b), indicating activation of autolysosome induction to reduce Aβ plaques in the AD brain. Therefore, the use of hNTSCs to clear Aβ deposits by activating autophagic regulation would be a promising therapeutic strategy in AD.

A main feature related to aging is cognitive decline. Recently, it was discovered that brain expression of TIMP2, which is a blood-borne factor enriched in human cord plasma, young mouse plasma, and the hippocampus, declines with age [52]. Injection of TIMP2 into aged mice revitalized the hippocampus, with improvement in neural plasticity and cognitive benefits, suggesting the relevance of TIMP2 expression levels in the AD brain to cognitive recovery. Notably, our data demonstrated the relevance of cognition and TIMP2 levels in the mouse brain in the AD model. Tg-hNTSC mice showed significantly higher levels of TIMP2 in the brain (Fig. 5d, e), supporting the much greater improvement in cognitive function observed in Tg-hNTSC mice than in Tg-sham or Tg-hBMSC mice, as shown in Fig. 5a–c. Moreover, it seems that higher levels of TIMP2 in the brains of Tg-hNTSC mice than in those of Tg-hBMSC mice were a result of greater levels of TIMP2 in hNTSCs than hBMSCs in culture (Fig. 5f). Cognitive capacity has been related to neuron number and function in the brain. We found a significant increase (1.6-fold) in the number of neurons within cortical regions of the brain in Tg-hNTSC mice compared with Tg-sham mice at 7 weeks after transplantation (Fig. 6a–c). Importantly, a great number of human cells were observed around the injection site in the brains of Tg-hNTSC mice. At 7 weeks after transplantation, approximately 2.5-fold more human cells were detected around the stereotaxic injection site in the brains of Tg-hNTSC mice than in those of Tg-hBMSC mice (Fig. 6d, e). Of course, many hNTSCs engrafted into the brain differentiated to express neuron proteins at 7 weeks after transplantation. Approximately 3.0-fold more cells in Tg-hNTSC mice were double positive for HuNu and NeuN than in those of Tg-hBMSC (Fig. 6f–h), suggesting a greater improvement in the cognitive deficit in Tg-hNTSC mice than in Tg-hBMSC mice. These data suggest that hNTSCs could overcome the limitations of using MSCs, which have low engraftment, as they have the ability to differentiate into neurons to replace damaged neurons after transplantation.

Here, we showed the feasibility of hNTSCs for use in AD treatment. hNTSCs demonstrated great cell growth and neural properties in vitro. In addition, transplantation of hNTSCs showed greater therapeutic potential than transplantation of hBM-MSCs in an AD mouse model. Taken together, our data clearly indicate that hNTSCs greatly reduce AD neuropathology and improve cognitive function. This reliable evidence supports the conclusion that hNTSCs are a valuable cell source for the treatment of patients with AD. In the future, research using a sporadic AD model may improve the potential therapeutic value of hNTSCs for patients in need of treatment for AD.

There are many risk factors associated with various pathways influencing AD development. Recent studies have shown the possibility of different treatment approaches based on the pathogenesis of AD not only using combination therapies including Aβ and tau but also considering insulin and cholesterol metabolism, vascular function, synaptic plasticity, and epigenetics [94–98]. The combination of therapeutic approaches has great potential to alter AD progression. Moreover, the importance of novel biomarker detection lies in the possibility that AD interventions represent valuable lines of research. The new genes and molecules hold the potential to allow us to categorize AD subtypes based on the clinical history of the patient, improve predictions about the evolution of the disease, and eventually choose a more personalized therapeutic strategy.

## Conclusions

This study showed the clinical feasibility of hNTSCs as a potent alternative source of stem cells for translation applications in treating AD and demonstrated specific benefits in vitro and in vivo. hNTSCs can be easily isolated from inferior turbinate tissue removed during turbinate resection via minimally invasive collection procedures. hNTSCs showed a high proliferative capacity and great neurogenic properties in vitro. Notably, transplantation of hNTSCs greatly reduces AD neuropathology and improves cognitive function than transplantation of hBM-MSCs in an AD mouse model. These findings provide reliable evidence supporting the clinical application of hNTSCs for use in AD treatment.

## Data Availability

The datasets generated during and/or analyzed during the current study are available from the corresponding author on reasonable request.

## Abbreviations

Aβ: Amyloid beta
AD: Alzheimer’s disease
BM-MSCs: Bone marrow-derived mesenchymal stem cells
DAPI: 4’,6-Diamidino-2-phenylindole
DMEM: Dulbecco’s modified Eagle’s medium
FAD: Familial Alzheimer’s disease
FBS: Fetal bovine serum
HuNu: Human nuclei
α-MEM: α-Minimum essential medium
NCSCs: Neural crest-derived stem cells
NEP: Neprilysin
NTSCs: Neural crest-derived nasal turbinate stem cells
PBS: Phosphate-buffered saline
PET/CT: Positron emission tomography-computed tomography
PFA: Paraformaldehyde
SSEA-3: Stage-specific embryonic antigen-3
TG: Transgenic
TIMP2: Tissue inhibitor of metalloproteinases 2
TLR: Toll-like receptor
UCB-MSCs: Umbilical cord blood-derived mesenchymal stem cells
WT: Wild-type
